# Measurement of body composition in postpartum South African women living with and without HIV infection

**DOI:** 10.3389/fnut.2024.1280425

**Published:** 2024-02-07

**Authors:** Hlengiwe P. Madlala, Landon Myer, Hayli Geffen, Demi Meyer, Amy E. Mendham, Julia H. Goedecke, Angela M. Bengtson, Jennifer Jao, Lara R. Dugas

**Affiliations:** ^1^Division of Epidemiology and Biostatistics, School of Public Health, University of Cape Town, Cape Town, South Africa; ^2^Riverland Academy of Clinical Excellence (RACE), Riverland Mallee Coorong Local Health Network, South Australia Health, Berri, SA, Australia; ^3^Division of Physiological Sciences, Department of Human Biology, Faculty of Health Sciences, Health Through Physical Activity, Lifestyle and Sport Research Centre (HPALS), FIMS International Collaborating Centre of Sports Medicine, University of Cape Town, Cape Town, South Africa; ^4^Biomedical Research and Innovation Platform, South African Medical Research Council, Cape Town, South Africa; ^5^Department of Epidemiology, Rollins School of Public Health, Emory University, Atlanta, GA, United States; ^6^Division of Infectious Diseases, Department of Paediatrics, Northwestern University Feinberg School of Medicine, Chicago, IL, United States; ^7^Division of Infectious Diseases, Department of Medicine, Northwestern University Feinberg School of Medicine, Chicago, IL, United States; ^8^Public Health Sciences, Parkinson School of Health Sciences and Public Health, Loyola University Chicago, Maywood, IL, United States

**Keywords:** body composition, women with HIV, postpartum, dual-energy X-ray absorptiometry (DXA), bioelectrical impedance analysis (BIA), air displacement plethysmography (ADP)

## Abstract

**Background:**

While several methodologies are available to measure adiposity, few have been validated in sub-Saharan African (SSA) and none in postpartum African women living with HIV (WLHIV). We compared bioelectrical impendence analysis (BIA) and air displacement plethysmography (ADP) against dual *x*-ray absorptiometry (DXA) in South African women and examined differences by HIV and body mass index (BMI) status.

**Methods:**

Lin’s concordance correlation coefficient (CCC) test was used to examine fat mass (FM), fat free mass (FFM), and total body fat percent (%BF) difference between BIA vs. DXA, and ADP vs. DXA in women living with HIV (*n* = 57) and without HIV (*n* = 25). The Bland Altman test was used to assess mean differences and the direction of bias.

**Results:**

The median age was 31 years (IQR, 26–35) and months postpartum were 11 (IQR, 7–16), 44% of the women had obesity. Lin’s CCC for BIA and ADP vs. DXA were both 0.80 for %BF and 0.97 for FM, and 0.86 and 0.80 for FFM, respectively. Mean differences (DXA-BIA and ADP estimates) were 0.22 ± 4.54% (*p* = 0.54) and 3.35 ± 3.27% (*p* < 0.01) for %BF, −0.82 ± 3.56 kg (*p* = 0.06) and 1.43 ± 2.68 kg (*p* = 0.01) for FM, −1.38 ± 3.61 kg (p = 0.01) and − 3.34 ± 2.37 kg (*p* < 0.01) for FFM, respectively. BIA overestimated %BF in WLHIV and underestimated it in women with obesity.

**Conclusion:**

Body composition measurements using BIA and ADP correlated well with DXA, thereby providing alternative, safe tools for measuring postpartum FM and FFM in SSA women, including WLHIV.

## Introduction

Pregnancy and the postpartum period (after childbirth) are recognised as a future window for metabolic health, including long-term obesity and subsequent risk for non-communicable diseases (NCDs) (1). However, data for postpartum body composition amongst Sub-Saharan Africa (SSA) women of African origin, including women living with HIV (WLHIV) are lacking. SSA has the highest rates of HIV worldwide, with women of reproductive age being predominately affected (2). Historically, weight loss was a common symptom in people living with HIV. However, with the recent policy shift to immediate treatment upon HIV diagnosis, there are increasing concerns about high levels of obesity and NCDs in people living with HIV (3). Indeed, in a recent study of Black South African WLHIV compared to those without HIV, it was found that irrespective of HIV status, women gained rather than lost weight at 12 months postpartum (4). However, it has been shown that body composition may differ by HIV status, with altered body fat partitioning (5, 6), as well as the long-term consequences to gaining differential fat mass (FM) or fat free mass (FFM), implicated in long-term metabolic health outcomes (7).

While dual-energy X-ray absorptiometry (DXA) is currently the gold standard for the analyses of FM and FFM, exposure to radiation and the requirement for a radiographer create significant limitations in low-income settings. Alternatively, bioelectrical impedance (BIA) and air displacement plethysmography (ADP) are safe and rapid methodologies that have been validated against DXA in other settings (8, 9). As with DXA, both BIA and ADP provide detailed body composition data including FM and FFM, which are more closely associated with health outcomes than anthropometry and body mass index (BMI) (10, 11). Increased FM is a risk factor for adverse maternal health outcomes, including diabetes and cardiovascular disease (CVD) (12). In contrast, FFM is a metabolically active tissue that is associated with reduced risk of metabolic disorders, including reduced mortality (7, 13). Accordingly, differentiating between FM and FFM using a safe and simple method in low-resourced communities can assist with risk stratification to help direct limited intervention resources to high-risk groups. Therefore, the goal of this study was to compare BIA and ADP against DXA in South African women within the postpartum period and examine differences by HIV and BMI status.

## Methods

### Study participants

A convenience sample of 82 South African women (aged ≥18 years) who were followed through in a larger study (*n* = 250) study of cardiometabolic health complications, were recruited between May 2021 and June 2022 and evaluated between 6 and 25 months postpartum. Women resided in Gugulethu, a low-income urban informal township, which is part of the greater Cape Town metropole, and home to almost 300,000 people of predominantly black African ethnic group (98.8%) (14). WLHIV were on ART for at least 1 year; and were either using efavirenz- (tenofovir 300 mg + emtricitabine 200 mg/lamivudine 300 mg + efavirenz 600 mg) or dolutegravir-based ART (tenofovir 300 mg + emtricitabine 200 mg/lamivudine 300 mg + dolutegravir 50 mg) provided as a fixed-dose combination pill taken once daily. Anthropometry and body composition assessment using the three methods (BIA, ADP, and DXA) were conducted on the same day in fasted (10 h) participants. All study procedures were reviewed and approved by the Faculty of Health Sciences Human Research Ethics Committee of the University of Cape Town (HREC 653/2020), and all participants signed a written informed consent explained in their local isiXhosa language prior to participation.

### Anthropometry

Weight (kg) and height (cm) measurements were examined in light clothing and no shoes using a calibrated scale (Charder, Taichung City, Taiwan) accurate to within 0.2 kg and a stadiometer (Seca, Birmingham, United Kingdom) accurate to within 0.1 cm. BMI was then calculated as weight divided by height squared; and categorised based on World Health Organisation criteria as underweight (<18.5), normal (18.5–24.9), overweight (25–29.9) and obese (≥30) in kg/m^2^ (15).

### Body composition

Three methods of whole-body composition were used to measure total body FM and FFM reported in absolute measures (kg). Total body FM is also presented relative to total body mass and reported as a percentage (%BF).

### Dual-energy X-ray absorptiometry

The participants were scanned using a low intensity *x*-ray beam by the study radiologist, while lying in a supine position wearing a laboratory gown with no jewellery. DXA scans (Hologic Discovery-W [S/N 71201], Bedford, MA, United States) were analysed using APEX software version 12.7.3.7 (16). *In vivo* precision was previously determined in our laboratory for FFM (0.7%) and FM (1.67%) by measuring 30 individuals twice on the same day with repositioning (17). The DXA unit was calibrated daily using a spine phantom, and participants received approximately 8.3 micro-Sieverts of radiation, considerably less than a transcontinental flight (60 micro-Sieverts).

### Bioelectrical impedance

A single-frequency (50 kHz) impedance analyser (model BIA 101Q; RJL Systems, Clinton Township, MI, United States) was used to obtain measures of resistance and reactance. Following this, the measured whole-body electrical resistance, reactance, height, weight and sex were entered into a BIA-specific equation, previously validated in Black South African women (18), and used to estimate measures of FM and FFM. The BIA unit was calibrated weekly using a test resistor.

### Air displacement plethysmography

Participants wore tight-fitting clothing and a cap, and were tested for body and lung volumes using the BodPod system (Cosmed, Rome, Italy). Body weight and volume assessments were conducted and corrected for thoracic gas volume (TGV) using predicted values. The ORTIZ equation for African descent population was used to calculate FM and FFM based on density and volume (19). The BodPod System is calibrated daily using a two-point calibration procedure.

### Statistical analysis

All statistical analyses were performed using STATA version 14 (Statacorp, Texas, United States) and figures were generated using R (R Foundation, Vienna, Austria). An alpha *p* value of 0.05 was used to denote statistical significance. Participant characteristics were summarised and presented as median (25–75th percentile) for continuous and *n* (%) for categorical variables. Characteristics stratified by HIV status were compared using Wilcoxon rank-sum test for continuous variables, significance was set at *p* < 0.05. Lin’s concordance correlation coefficient (CCC) method (95% CI) was used to test the difference between BIA and ADP against DXA for the assessment of %BF, FM, and FFM. This method assesses both precision and accuracy to determine how far the observed data deviate from the line of perfect concordance (20, 21). The range is −1 to 1, with 1 being the perfect agreement between two measures. Next, we applied the Bland Altman test to examine the differences between the measures and to understand the direction of the bias (22) in the overall sample as well as in sub-groups stratified by HIV, BMI and months postpartum. To calculate the mean differences for BIA and ADP against DXA, BIA and ADP measurements were subtracted from those obtained using the DXA scan. Heteroscedasticity was tested to examine a linear relationship between the mean difference and %BF, FM and FFM.

## Results

### Participant characteristics

Of the 82 participants, 78 (95%) had both BIA and DXA, while 53 (65%) had both ADP and DXA assessments. The median age was 31 years (IQR, 26–35) and time postpartum was 11 months (IQR, 7–16), few (18%) women were primigravid and 70% were living with HIV (Supplementary Figure 1). Overall, the median weight was 75.9 kg (IQR, 66.2–90.8) and BMI was 28.9 kg/m^2^ (IQR, 25.1–34.9). Women with HIV were lighter (72.5 kg [IQR, 63.3–87.3] vs. 88.8 kg [IQR, 73.8–96.1], *p* = 0.03) and had lower BMI (28.3 kg/m^2^ [IQR 23.4–32.8] vs. 32.8 kg/m^2^ [IQR 27.6–36.7], *p* = 0.03) compared to women without HIV. 44% women had obesity, 52% of which were women without HIV, and 40% were WLHIV (*p* = 0.22).

### DXA body composition

Using DXA, overall body composition was 46.2% (IQR 42.9–50.7) for %BF, 33.9 kg (IQR 27.1–44.4) for FM and 39.8 kg (IQR 35.3–43.4) for FFM (Supplementary Figure 1). Compared to women without HIV, WLHIV had significantly lower FM (31.9 vs. 40.9 kg, *p* = 0.02) and FFM (39.3 vs. 41.7 kg, *p* = 0.01), which was driven by their overall lower body weights.

### BIA vs. DXA

**Figure 1:**
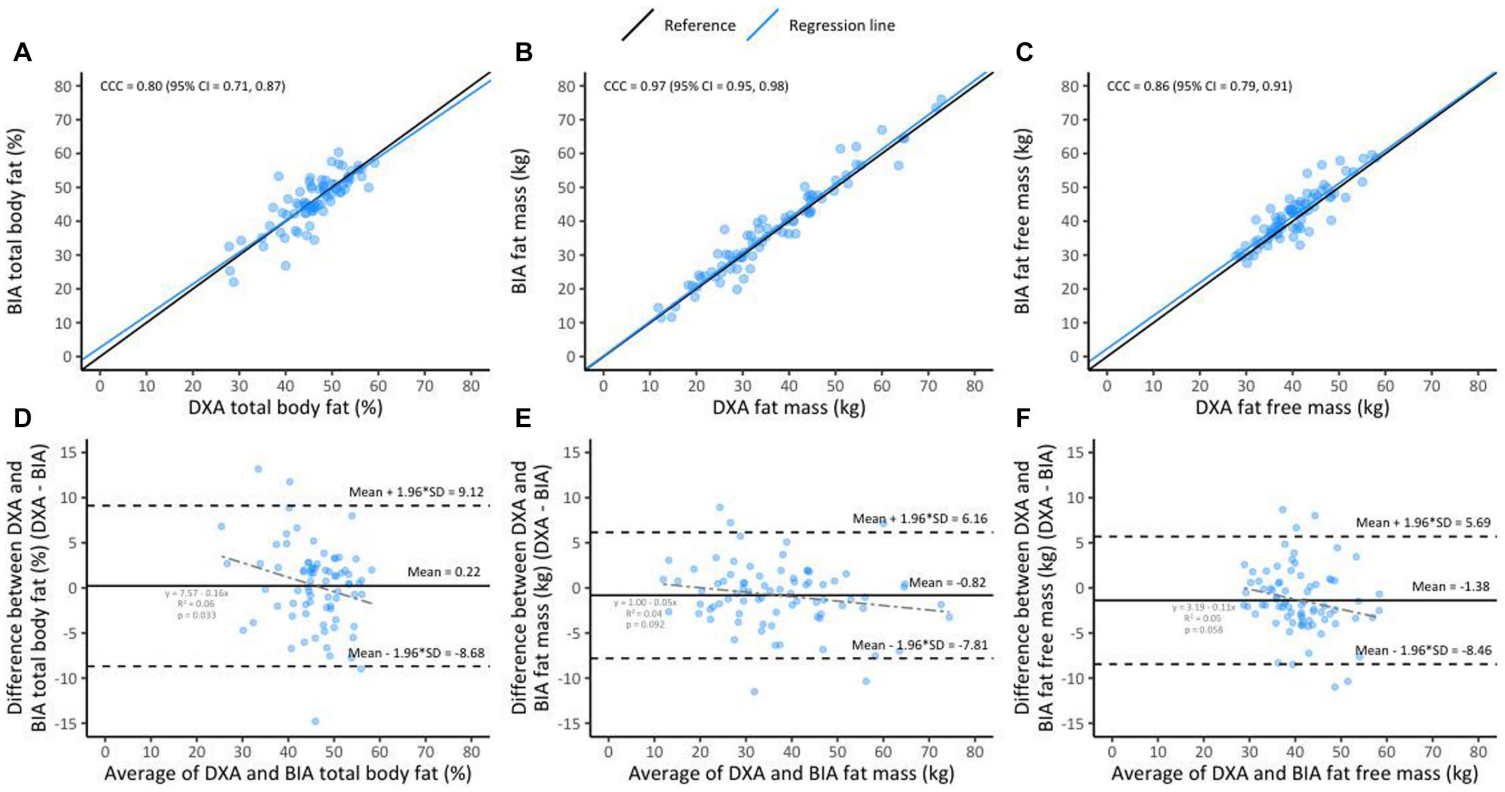
Lin’s concordance between BIA and DXA in the overall sample (*n* = 78, **A–C**); the black line indicates perfect concordance while the blue line indicates the observed relationship between BIA and DXA for %BF **(A)**, FM **(B)**, and FFM **(C)**. Bland Altman limit of agreement between BIA and DXA **(D–F)**; the solid line indicates the mean difference between BIA and DXA for %BF **(D)**, FM **(E)**, and FFM **(F)**. The area inside the dotted lines indicate 95% limits of agreement (HIV+ [*n* = 54] and HIV− [*n* = 24]).

**Figure 2:**
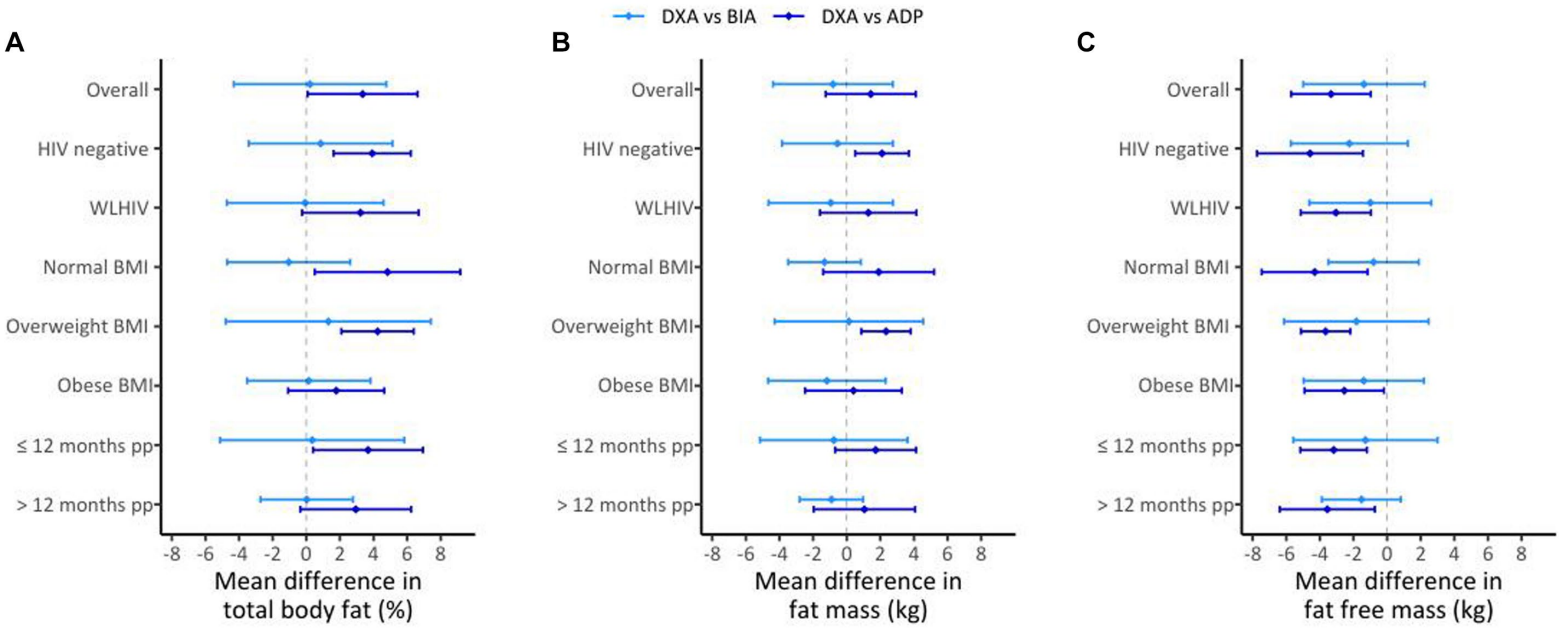
Mean differences for DXA vs. BIA (light blue lines) and DXA vs. ADP (dark blue lines) in the overall sample (*n* = 82, **A–C**) and by HIV, BMI, and postpartum (pp) duration; for %BF **(A)**, FM **(B)**, and FFM **(C)**. The diamond represents the estimate of the mean difference for the comparisons and the bars represent the mean difference +/− the estimated SD of the mean difference.

Lin’s CCC for BIA vs. DXA was 0.80 (95% CI 0.71–0.87) for %BF, 0.97 (95% CI 0.95–0.98) for FM and 0.86 (95% CI 0.79–0.91) for FFM (Figures 1A–C). The mean differences (DXA minus BIA) were 0.22 ± 4.54% (*p* = 0.54) for %BF, −0.82 ± 3.56 kg (*p* = 0.06) for FM and − 1.38 ± 3.61 kg (*p* = 0.01) for FFM (Figures 1D–F). Overall, while differences were considered small and not clinically meaningful, %BF measurements using BIA were consistently lower than %BF measured using DXA, but that both FFM and FM BIA measurements were higher compared to DXA. Notably, the mean difference decreased with increasing %BF (*r^2^* = 0.06, *p* = 0.03), although this was not the case for FM and FFM. The direction of bias for %BF (indicated by positive or negative sign of the mean difference) was different in HIV and BMI sub-groups (Figure 2A, light blue lines and Supplementary Figure 2). %BF derived using BIA was higher for WLHIV (mean difference − 0.07 ± 4.66%) but lower for women without HIV (mean difference 0.85 ± 4.28%). This result is likely driven by BMI differences between the two groups (28.3 vs. 32.8 kg/m^2^, *p* = 0.03; Supplementary Figure 1). Similarly, %BF using BIA was on average higher for women with normal BMI (mean difference − 1.05 ± 3.66%) but lower for women with overweight (mean difference 1.31 ± 6.10%) and obesity (mean difference 0.15 ± 3.67%) (Figure 2A, light blue lines and Supplementary Figure 2). A difference in the direction of bias was also observed in BMI sub-groups for FM measurements. Using BIA, FM was higher for women with normal BMI (mean difference − 1.32 ± 2.16 kg) and obesity (mean difference − 1.18 ± 3.49 kg) but lower for women with overweight (mean difference 0.13 ± 4.42 kg) (Figure 2B, light blue lines and Supplementary Figure 2). Postpartum months (≤12 vs. >12 months) did not affect the direction of the bias for all BIA vs. DXA measurements.

### ADP vs. DXA

**Figure 3:**
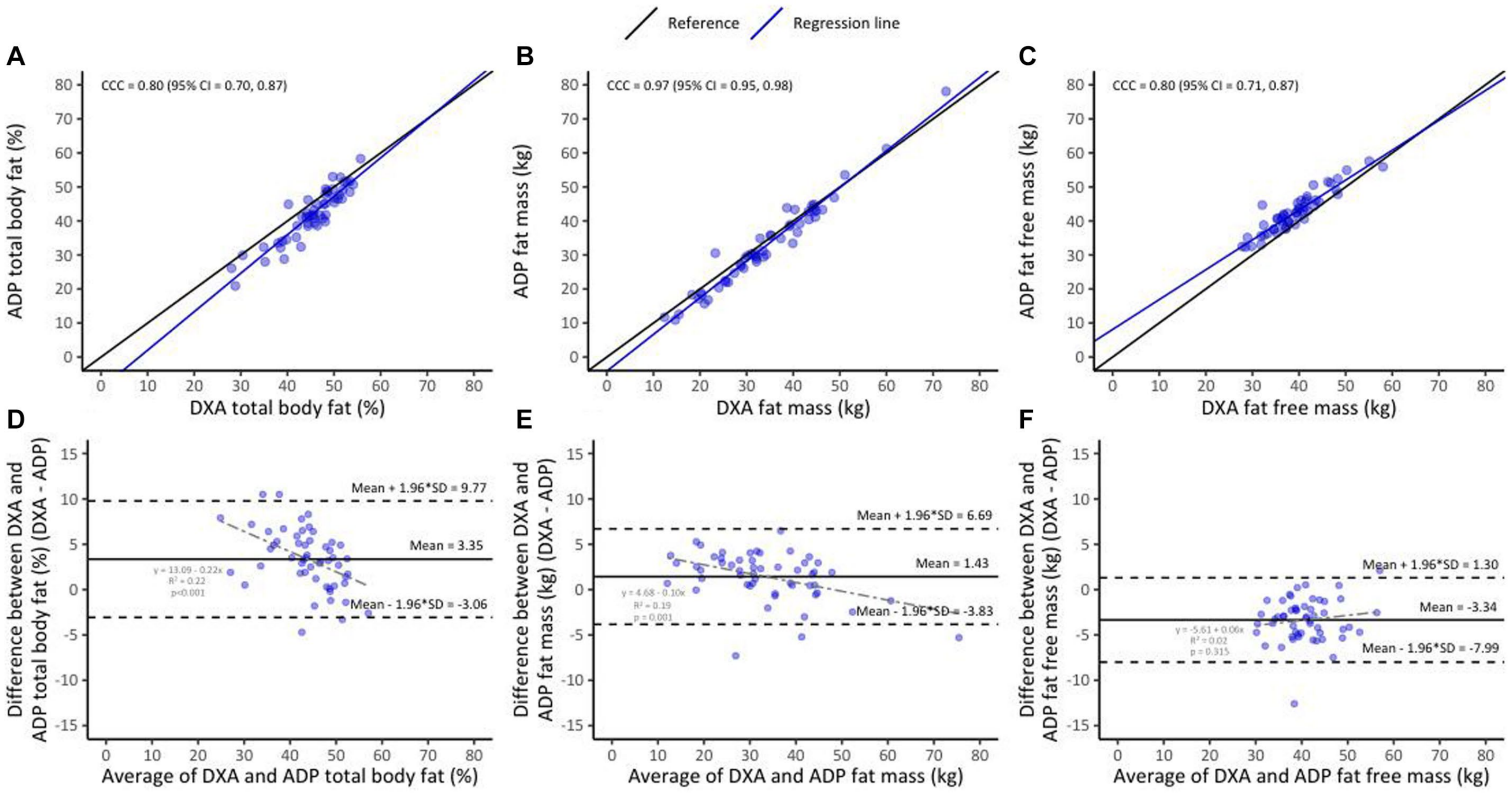
Lin’s concordance between ADP and DXA in the overall sample (*n* = 53, **A–C**); the black line indicates perfect concordance while the purple line indicates the observed relationship between ADP and DXA for %BF **(A)**, FM **(B)**, and FFM **(C)**. Bland Altman limit of agreement between BIA and DXA **(D–F)**; the solid line indicates the mean difference between ADP and DXA for %BF **(D)**, FM **(E)**, and FFM **(F)**. The area inside the dotted lines indicate 95% limits of agreement (HIV+ [*n* = 43] and HIV− [*n* = 10]).

Lin’s CCC was 0.80 (95% CI 0.70–0.87) for %BF, 0.97 (95% CI 0.95–0.98) for FM and 0.80 (95% CI 0.71–0.87) for FFM (Figures 3A–C). The mean difference (DXA minus ADP) was 3.35 ± 3.27% (*p* < 0.01) for %BF, 1.43 ± 2.68 kg (*p* = 0.01) for FM and − 3.34 ± 2.37 kg (*p* < 0.01) for FFM (Figures 3D–F). This shows that %BF and FM measurements completed using ADP were lower than those completed using DXA, but that FFM ADP measurements were higher compared to DXA measurements. The mean difference decreased with increasing %BF (*r^2^* = 0.22, *p* < 0.01) and FM (*r^2^* = 0.19, *p* = 0.01) but not FFM. The direction of this bias (represented by the positive or negative sign of the mean difference) for ADP did not differ between sub-groups of HIV, BMI or postpartum months (Figures 2A–C, dark blue lines).

## Discussion

To our knowledge, this is the first study to examine body composition measurements comparing BIA and ADP against the gold-standard DXA in postpartum WLHIV and women without HIV in SSA. Using Lin’s CCC, we found that the agreement for both BIA and ADP with the gold standard test was overall high, but with some variation depending on HIV and BMI status but not postpartum months.

Bioelectrical impendence analysis is a portable, quick and inexpensive measure of body composition based on the electrical resistance principle. We found that BIA assessments were highly correlated with DXA, especially for FM (*r* = 0.97). FM is associated with increased risk of adverse metabolic health including a lifetime risk of CVDs (23). However, changes in FM may not always be reflected through postpartum weight, hence, body composition assessment is a superior measure. This was shown in a study by Cho and Junamala, who found that FM was increased in postpartum women despite lack of changes in weight (24, 25). The mean differences between BIA and DXA were between 1 and 3% for %BF, FM and FFM. While there are no established criteria for acceptable differences, this difference is considerably low. Therefore, these results suggest that BIA is a good measure of adiposity in this population. Indeed, BIA body composition estimation equations used in this study were validated against doubly labelled water method in populations of African origin, including South African women (18). However, we noted that in the overall sample, BIA underestimated %BF, albeit by a mean of 0.22%. When stratified by HIV and BMI status, BIA overestimated %BF amongst WLHIV and underestimated it amongst women with overweight and obesity. This observation of HIV group differences is likely driven by BMI differences, and both the HIV and BMI comparisons are in agreement as they indicate that the mean difference decreases with increasing %BF. There was no bias observed in the FFM in kg.

Air displacement plethysmography is a rapid, non-invasive but relatively costly new technology for assessment of body composition (26). Like BIA, we found that ADP assessments were highly correlated with DXA, especially for FM (*r* = 0.97). The mean differences between ADP and DXA assessments ranged between 4 and 8% for %BF, FM and FFM. The women included in the development of the ORTIZ equation used to estimate body composition amongst African descent populations were African Americans (27), with no representation of SSA women. As a result, we speculate that this may have contributed to the observed larger percentage error of ADP and that the development of an equation using SSA women might improve measurement precision. ADP underestimated %BF and FM, and overestimated FFM. However, with increasing adiposity, the mean difference decreased. Overestimation of FFM and underestimation of the fat content by ADP compared to DXA was also reported in other studies (28, 29). Obesity above 40 kg/m^2^ has been shown to invert the ADP bias direction resulting in underestimation of FFM and overestimation of fat content (28). This would be an important consideration for our population as there is a high prevalence of overweight and obesity (64%) in South African women (30).

Our study is not without limitations. Firstly, we note that in our study there was variation in the timing of the postpartum visit attendance, which may have led to increased variability in the adiposity observed. This variability was however, mitigated by completing all the body composition assessments on the same day, allowing participants to act as their own controls. Secondly, we had fewer women enrolled without HIV. On the other hand, our study exploring body composition during the postpartum period, using the gold standard technique is a significant strength. In conclusion, we show that body composition assessment’s using both BIA and ADP correlated well with DXA, offering alternative tools for measuring postpartum body composition. Implementation of such tools for routine monitoring of FM and FFM would be an important step towards achieving WHO sustainable development goal 3 target of one third reduction in premature mortality from NCDs by 2030.

## Data availability statement

The raw data supporting the conclusions of this article will be made available by the authors, without undue reservation.

## Ethics statement

The studies involving humans were approved by Faculty of Health Sciences Human Research Ethics Committee of the University of Cape Town. The studies were conducted in accordance with the local legislation and institutional requirements. The participants provided their written informed consent to participate in this study.

## Author contributions

HM: Conceptualization, Data curation, Funding acquisition, Investigation, Methodology, Writing – original draft. LM: Conceptualization, Methodology, Supervision, Writing – review & editing, Investigation. HG: Formal Analysis, Writing – review & editing. DM: Data curation, Project administration, Writing – review & editing. AM: Data curation, Writing – review & editing. JG: Conceptualization, Resources, Writing – review & editing, Supervision. AB: Conceptualization, Writing – review & editing. LD: Conceptualization, Investigation, Methodology, Supervision, Writing – review & editing.
