# Supplementary figures and images for: Measurement of body composition in postpartum South African women living with and without HIV infection
Source: Front Nutr. 2024 Feb 7;11:1280425. doi: 10.3389/fnut.2024.1280425 (PMC10879415; doi:10.3389/fnut.2024.1280425)

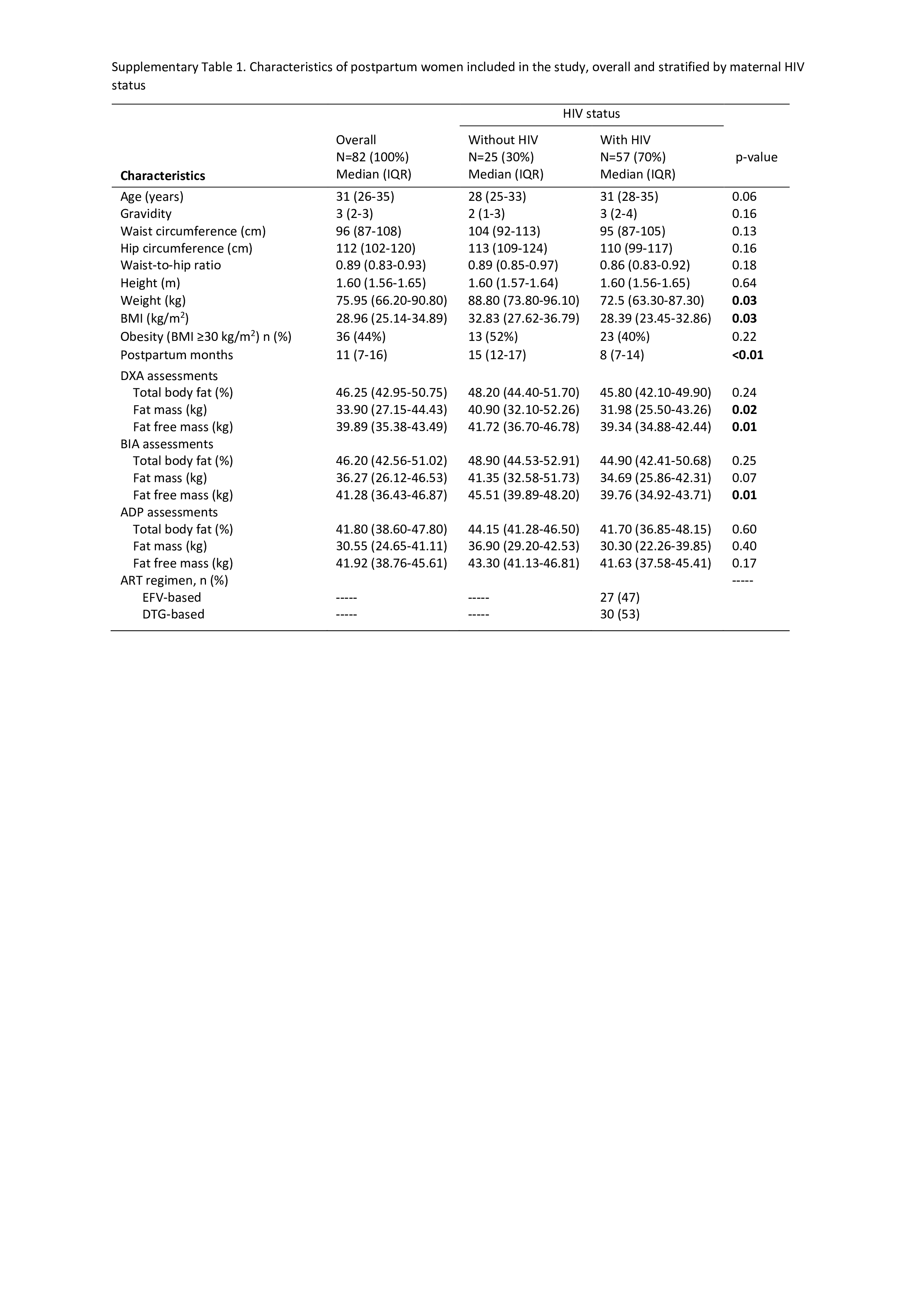

Supplement: Supplementary file 1 [file Image_1.jpg]

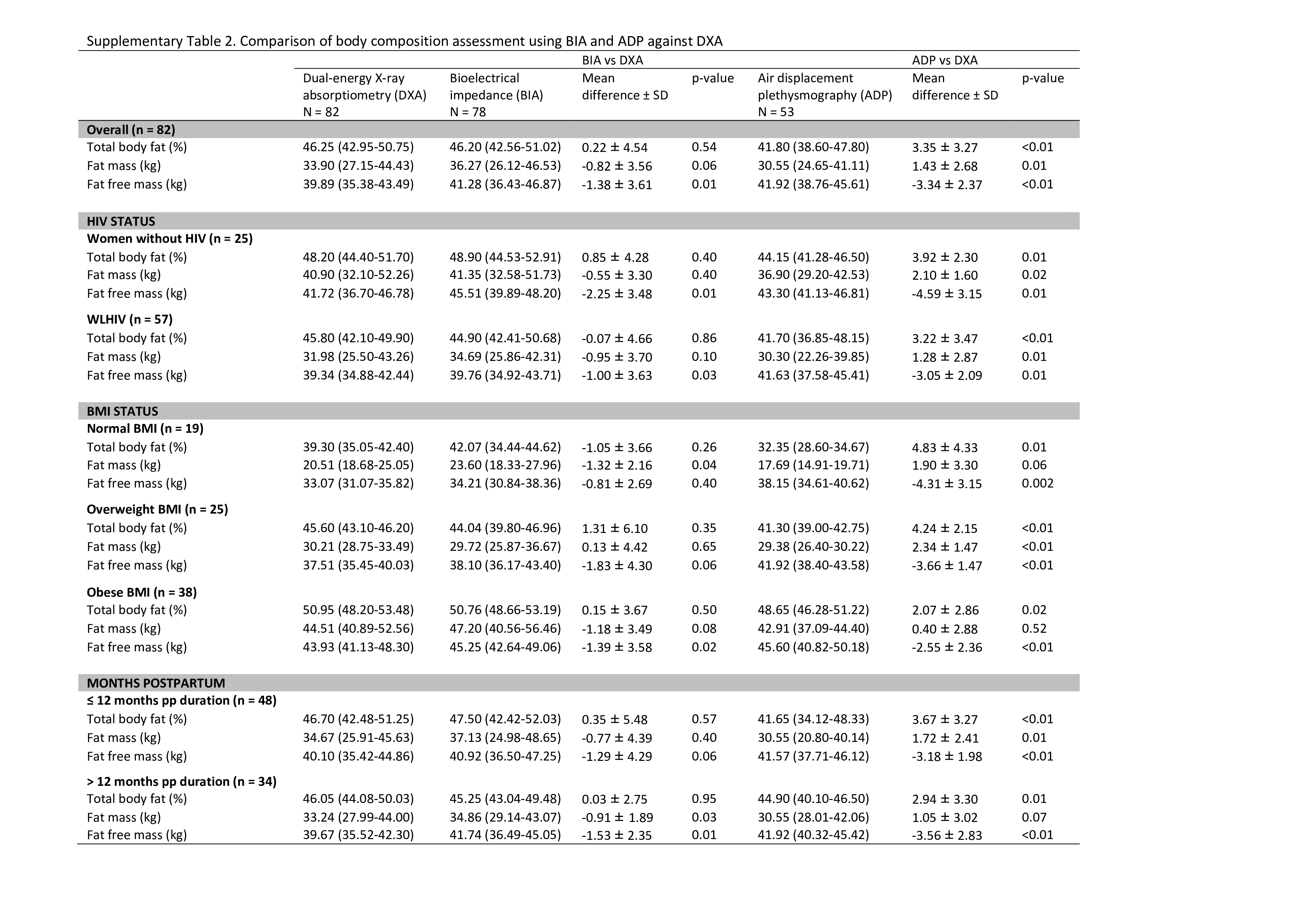

Supplement: Supplementary file 2 [file Image_2.jpg]
